# Multimodal quantitative magnetic resonance imaging alterations of the basal ganglia circuit underlie the severity of bulimia nervosa

**DOI:** 10.1016/j.ijchp.2025.100557

**Published:** 2025-03-13

**Authors:** Yiling Wang, Lirong Tang, Weihua Li, Miao Wang, Qian Chen, Fengxia Yu, Zhenghan Yang, Zhanjiang Li, Zhenchang Wang, Jiani Wang, Guowei Wu, Peng Zhang

**Affiliations:** a Department of Radiology, Beijing Friendship Hospital, Capital Medical University, No.95 Yongan Road, Xicheng District, Beijing, 100050, China; b Beijing Anding Hospital Capital Medical University, No.5 Ankang Lane, Dewai Avenue, Xicheng District, Beijing, 100088, China; c The National Clinical Research Center for Mental Disorders & Beijing Key Laboratory of Mental Disorders, Beijing Anding Hospital, Capital Medical University, No.5 Ankang Lane, Dewai Avenue, Xicheng District, Beijing, 100088, China; d Peking University, No.5 Summer Palace Road, Haidian District, Beijing, 100871, China; e CAS Key Laboratory of Behavioral Science, Institute of Psychology, Chinese Academy of Sciences, No.16 Lincui Road, Chaoyang District, Beijing, 100020, China

**Keywords:** Bulimia nervosa, Severity, Basal ganglia circuit, Functional connectivity, White matter integrity

## Abstract

**Background:**

Neuroimaging alterations in the basal ganglia circuit have been reported to correlate with the severity of various eating or addictive disorders, but their relationship to the severity of bulimia nervosa (BN) remains largely unknown. This study sought to investigate the basal ganglia circuit structural and functional imaging differences in BN patients with different severity.

**Methods:**

Based on the MRI data acquired from 34 mild BN patients, 35 moderate-to-extreme BN patients and 35 healthy controls (HCs), differences in gray matter volume (GMV), fractional anisotropy, fractional amplitude of low-frequency fluctuation (fALFF), and seed-based functional connectivity (FC) of basal ganglia circuit (including the caudate, globus pallidus, nucleus accumbens and putamen) were compared across the three groups.

**Results:**

Compared to HCs, the mild patients only exhibited decreased fALFF in the left ventromedial putamen and increased FC between the nucleus accumbens and orbitofrontal cortex, without any structural imaging alterations. Whereas, the moderate-to-extreme patients exhibited significant basal ganglia imaging alterations, characterized by widespread higher FC between basal ganglia regions and several frontal-parietotemporal regions, and disrupted white matter integrity. Based on receiver operating characteristic curves, we discovered that seed-based FC had acceptable discriminatory values in classifying BN patients into mild or moderate-to-extreme groups.

**Conclusion:**

This study reveals that basal ganglia circuit imaging alterations in BN patients become more pronounced with increasing disease severity, suggesting a crucial role of basal ganglia circuit in the progression of BN. Functional network reorganization between basal ganglia and other regions may serve as a potential risk imaging marker for BN progression.

## Introduction

Bulimia nervosa (BN) is an eating disorder featured with recurrent episodes of uncontrolled overeating and inappropriate compensatory behaviors aimed at preventing weight gain (Treasure, Duarte and Schmidt, 2020). It is considered to be related to serious medical complications, suicidal risk, psychosocial impairment, and socioeconomic burden (van Eeden, van Hoeken & Hoek, 2021; Widiger & Hines, 2022). However, current frontline treatments for BN yield remission rates of only 30 %−40 %, posing a significant challenge in managing this disorder (Treasure et al., 2020). Disease severity is one of the most pivotal factors influencing the BN treatment outcome, and studies have shown that patients with mild BN have a better prognosis than those with moderate, severe, and extreme BN (Smith et al., 2017). Thus, improved identification and characterization of brain alterations associated with increased BN severity could help explore risk monitoring indicators and potential therapeutic targets.

According to the Diagnostic and Statistical Manual of Mental Disorders, fifth edition (DSM-5) criteria (American Psychiatric Association, 2013), the frequency of inappropriate weight compensatory behaviors defines the severity of BN, and such behavior is a major risk for increased mortality in patients with BN (Castillo & Weiselberg, 2017). Current theoretical models have proposed that inappropriate reward learning and habituation responses play a key role in the development and maintenance of BN patients’ inappropriate compensatory behavior (Cassioli et al., 2020; Smith & Graybiel, 2016; Steinglass, Berner & Attia, 2019). In addition, this behavior has also been linked to difficulties in emotion regulation and poor self-control (Berner & Marsh, 2014; McClure, Messer, Anderson, Liu & Linardon, 2022). The basal ganglia circuit, whose main component is the striatum (Mink, 1996), specifically consists of the caudate nucleus, putamen, nucleus accumbens, and pallidum (Ashby, Turner & Horvitz, 2010). This circuit engages in behavioral control, habit formation, emotion regulation, and reward processing (Gunaydin & Kreitzer, 2016; Kim & Hikosaka, 2015), which makes it a critical regulatory role in food intake and physical activity. Striatal regions, such as the caudate nucleus, the anterior part of the putamen, and the nucleus accumbens, have been known to be involved in reward-motivated decision-making and the acquisition of newly learned behaviors (Cox & Witten, 2019; Kelley, 2004). The pallidum and posterior part of the putamen are considered to help form rigid habits (Agustin-Pavon, Martinez-Garcia & Lanuza, 2014; Sommer, Costa & Hansson, 2014). Considerable neurochemical (Joshi, Schott, la Fleur & Barrot, 2022; Lin, Jan, Kydd & Russell, 2015; Raghanti et al., 2023) and animal research (Becchi et al., 2022; Smith et al., 2015) have uncovered the involvement of basal ganglia circuit in the onset and maintenance of various eating disorders and addictions.

In addition to the disease pathogenesis, prior studies have pointed out that neuroimaging alterations in the basal ganglia circuit are correlated with the severity of impulsive or compulsive behaviors in numerous eating or addictive disorders. Specifically, Haynos et al. (2021) have revealed that lower functional connectivity (FC) between the nucleus accumbens and prefrontal cortex is related to greater reversal of learning difficulties and frequency of overeating in individuals with binge eating disorder. Similarly, the severity of individuals with Internet gaming disorder has been reported to be associated with diminished frontostriatal FC (Dong et al., 2021). A previous study (Wang et al., 2022) found that the deformations of the subcortical basal ganglia structures in adolescents with substance use disorders were associated with the frequency of their impulsive behaviors. These findings led us to postulate a correlation between imaging alterations in the basal ganglia circuit and the severity of BN. Scholars have identified abnormalities in the basal ganglia circuit of BN patients via neuroimaging techniques (Berner et al., 2019; Coutinho et al., 2015; Wang et al., 2020). However, their precise relationship to disease severity remains largely unknown. Exploring the differences in the basal ganglia circuit imaging features in BN patients with mild and moderate-to-extreme severity may help to identify risk imaging markers for disease progression and potential therapeutic targets, further comprehending the role of the basal ganglia circuit in the BN neuropathological mechanisms.

In the present study, we divided BN patients into mild and moderate-to-extreme groups according to the severity specifier in DSM-5, and established a matched healthy control (HC) group. And we explored the structural (including gray matter volume and white matter integrity) and functional (including local neuronal activity and functional connectivity) imaging characteristics of basal ganglia circuit in patients with different severity using multimodal magnetic resonance imaging (MRI) technique. We hypothesized that imaging alterations in the basal ganglia circuit would be distinct for patients of different severity and that those with greater severity might exhibit more pronounced alterations compared with HCs.

## Methods and materials

This study was approved by the Ethics Committee of Beijing Friendship Hospital (Approval No 2021-P2–052–01) and conducted in accordance with the Declaration of Helsinki. Before the beginning of the study, the investigators informed the participants about the study procedures and signed a written informed consent document with them.

### Participants

Our initial study population consisted of 77 BN patients and 38 HCs. BN patients were recruited from outpatient services, while the HC group was recruited through internet advertisements. Since our study is part of an ongoing research series, these participants partially overlapped with our previous studies (Wang, Tang, Wang, Li et al., 2023; Wang, Tang, Wang, Wu et al., 2023). All of the participants were right-handed and matched for sex, age, and education level. The BN patients were recruited independently by two psychiatrists in the field of eating disorders, who made the diagnosis of BN using the Mini-International Neuropsychiatric Interview (MINI) 7.0.2 (Sheehan, 2016), a short structured interview compiled upon the DSM-5. Then, BN patients with a current diagnosis of a major psychiatric disorder (other than depression or anxiety), such as schizophrenia, bipolar disorder, dissociative disorder, or substance abuse, as well as those who had taken antipsychotics within two months prior to the experiment, were initially excluded from participation.

We included HCs who were of normal weight and who had no lifetime history of mental illness. Additionally, They underwent a MINI test to further confirm the absence of any potential psychiatric disorders.

The remaining exclusion criteria for all participants were consistent with our previous study (Wang, Tang, Wang, Li et al., 2023; Wang, Tang, Wang, Wu et al., 2023). The data from 5 BN participants were excluded from the subsequent analyses due to a history of alcohol abuse (*n* = 1), metal implants (*n* = 2), and claustrophobia (*n* = 2). And 3 HCs were excluded due to claustrophobia. After a rigorous screening process, a total of 72 BN patients and 35 HCs were included in the subsequent analyses.

### BN patient grouping

According to the DSM-5 (American Psychiatric Association, 2013), the severity specifier of BN is the patients' average weekly frequency of inappropriate weight compensatory behaviors over the past 3 months. To classify patients, we gathered data on the weekly frequency of compensatory behaviors for each patient during this period. Based on average weekly compensatory behavior frequency, patients were categorized into two groups: a mild BN group (1–3 episodes per week) and a moderate-to-extreme BN (≥ 4 episodes per week) group.

### Clinical assessment

All participants were asked to fast for at least four hours before receiving the MRI scans. Before the start of the scans, demographic information was collected from each subject. In addition, the disease duration of each patient was also collected. Subsequently, each subject completed six self-report questionnaires under the instruction of a specialized psychiatrist. The questionnaires included: (1). The Visual Analog Scale (VAS) (Blundell et al., 2010; Hill, Rogers & Blundell, 1995): this scale was employed to rate the subject's current level of hunger from 0 (not at all hungry) to 10 (very hungry); (2). The Chinese version of the Dutch Eating Behavior Questionnaire (DEBQ) (Wu, Cai & Luo, 2017): this scale was a 33-item scale consisting of 3 subscales and was utilized to assess subjects' restraint, emotional, and external eating behaviors; (3). The bulimia subscale of the Eating Disorders Questionnaire (EDI-BN) (Lee, Lee, Leung & Yu, 1997): this questionnaire was designed to evaluate subjects' bulimic drive; (4). The Eating Attitude Test (EAT-26) (Kang et al., 2017): this is a 26-item scale used to assess disordered eating attitudes and behaviors; (5) The Beck Depression Inventory (BDI) (Shek, 1990) and (6) the Self-Assessment of Anxiety Scale (SAS) (Zung, 1971): these two scales were used to measure subjects' depression and anxiety states, respectively. The Chinese version of these questionnaires has been validated to have good reliability and validity for evaluating eating disorders in Chinese people (Kang et al., 2017; Mo et al., 2020; Shek, 1990; Wu et al., 2017; Zung, 1971).

### MRI data acquisition

MRI data of all subjects were obtained using a 3.0-T MRI scanner (Prisma, Siemens, Erlangen, Germany), equipped with a 64-channel phased-array head coil. During the scan, subjects were asked to close their eyes and remain calm, awake, and keep their heads motionless. In addition, they were provided with earplugs and foam pads to minimize scanning noise and head movement. Initially, a conventional axial T2 sequence was obtained for each subject to screen for any visible brain abnormalities. Subsequently, high-resolution T1-weighted (T1w)structural images were collected in the sagittal position applying a three-dimensional magnetized preprocessed rapid acquisition gradient echo (MPRAGE) sequence, which were utilized to assess gray matter volume. Diffusion tensor imaging (DTI) for assessing white matter integrity was acquired using a single-shot gradient- echo-planar imaging (EPI) sequence, and resting-state functional MRI (rs-fMRI) data were obtained with the EPI sequence to assess local neuronal activity and functional connectivity. Detailed parameters of these sequences are listed in the Supplementary Material. Prior to the start of each sequence, subjects received an additional verbal reminder to ensure they remained awake throughout the scan. All participants confirmed that they did not doze off or fall asleep during the scanning process.

### Definition of the basal ganglia circuit

**Fig. 1:**
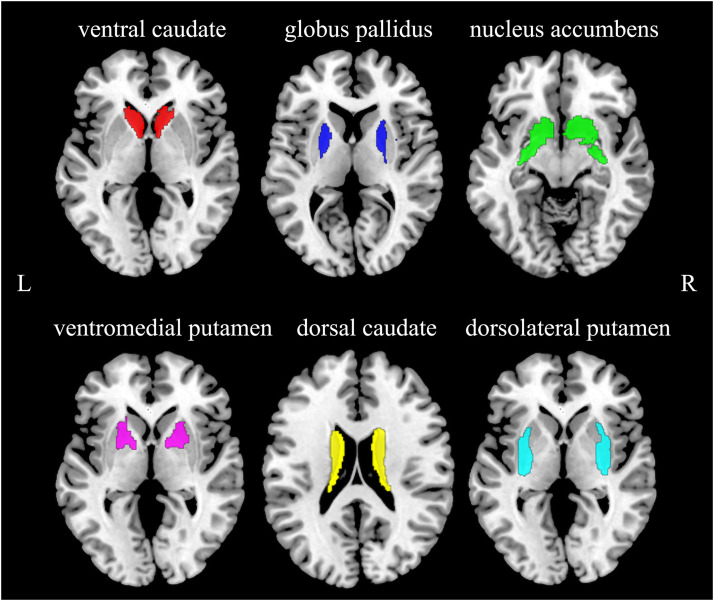
The regions of the basal ganglia circuit intersected by the Brainetome atlas.

The basal ganglia circuit was defined according to a publicly accessible atlas, namely Brainetome (Fan et al., 2016), which divided the basal ganglia into six regions: ventral caudate (vCAU), globus pallidus (GP), nucleus accumbens (NAc), ventromedial putamen (vmPu), dorsal caudate (dCAU), and dorsolateral putamen (dlPu) (Fig. 1). Then, the atlas was applied to all individual multimodal maps by spatially normalizing them into the atlas template space.

### Rs-fMRI data processing

#### Data preprocessing

The preprocessing of rs-fMRI data was conducted utilizing Data Processing & Analysis for Brain Imaging (DPABI: http://rfmri.org/dpabi) and Statistical Parametric Mapping (SPM12: http://www.fil.ion.ucl.ac.uk/spm) software that was installed in MATLAB 2013b. Its procedure was consistent with our previous study (Wang, Tang, Wang, Wu et al., 2023) and was detailed in the Supplementary Material. In this process, 3 BN subjects were excluded due to head movements exceeding the criteria (i.e., subjects with head movements > 2.0 mm translation and 2.0° rotation in any direction).

#### Calculation of fractional amplitude of low-frequency fluctuations (fALFF)

The fALFF calculation was also conducted applying DPABI software. First, a fast Fourier transform was employed to convert the preprocessed time series of each voxel into the frequency domain to obtain the power spectrum. Subsequently, the ratio of the power in the ALFF frequency range (0.01–0.08 Hz) to the full frequency range (0–0.25 Hz) was calculated to yield the fALFF. The fALFF value of each voxel was divided by the global mean ALFF value of each subject for standardization purposes. Lastly, the fALFF values for regions of the basal ganglia circuit were extracted in the DPABI tool for subsequent statistical analysis.

#### Calculation of seed-based FC

The seed-to-voxel FC calculations were conducted in the DPABI toolbox using the 12 regions of the basal ganglia circuit as seed points. The reference time series for each seed point was first obtained by averaging the time series of all voxels within each seed point. Next, the FC maps were obtained by calculating the correlation coefficient between the reference time series of each seed point and the time series of each voxel within the whole brain. Finally, Fisher r-to-z transformation was performed on the resulting FC maps to improve the normality.

### T1w structural data processing

The high-resolution T1w structural data were preprocessed using the Statistical Parametric Mapping 12 (SPM12)-based CAT12 package (http://dbm.neuro.uni-jena.de/cat/) in the MATLAB 2013b environment. Before data preprocessing began, we checked the data to exclude data with artifacts. We then preprocessed the data in a process consistent with our previous study (Wang, Tang, Wang, Wu et al., 2023), which mainly consists of three segments: (1) segmentation, (2) normalization, and (3) smoothing. The detailed steps were described in the Supplementary Material. Finally, based on the gray matter images generated by preprocessing, we used the Data Processing and Analysis for Brain Imaging (DPABI) toolbox (http://rfmri.org/dpabi) to extract the gray matter volume (GMV) value of each region in the basal ganglia circuit of each subject for the following statistical analysis.

### DTI data processing and white matter integrity property analysis

The preprocessing of diffusion-weighted imaging (DWI) data using QSIPrep to ensure high-quality, artifact-corrected datasets. For anatomical reference, T1w images were first corrected for intensity non-uniformity using the N4BiasFieldCorrection algorithm (ANTs 2.3.1) (Tustison et al., 2010) and used as the T1w-reference throughout the workflow. Skull stripping was performed using antsBrainExtraction.sh (ANTs 2.3.1) with the OASIS template as the target. The T1w-reference was then spatially normalized to the ICBM 152 Nonlinear Asymmetrical template (version 2009c, RRID) (Fonov, Evans, McKinstry, Almli & Collins, 2009) through nonlinear registration using antsRegistration (ANTs 2.3.1, RRID) (Avants, Epstein, Grossman & Gee, 2008). Segmentation of cerebrospinal fluid, white matter, and gray matter was performed using FAST (FSL 6.0.5.1, RRID) (Zhang, Brady & Smith, 2001) on the skull-stripped T1w images.

For DWI data, images with b-values <100 s/mm² were treated as *b* = 0 images. MP-PCA denoising was applied using MRtrix3’s dwidenoise (Veraart et al., 2016) with a 5-voxel window, followed by Gibbs unringing using mrdegibbs (Kellner, Dhital, Kiselev & Reisert, 2016). B1 field inhomogeneity was corrected via MRtrix3′s dwibiascorrect employing the N4 algorithm (Tustison et al., 2010), and the mean intensity of the DWI series was normalized across sequences. Motion and eddy current artifacts were addressed using FSL's eddy (version 6.0.5.1) (Andersson, Graham, Zsoldos & Sotiropoulos, 2016), which included outlier replacement, slice-based adjustments, and shell alignment. Framewise displacement (FD) and slicewise cross-correlation were calculated for further quality control. The preprocessed DWI data were resampled to ACPC space, resulting in 2 mm isotropic voxels for subsequent analysis.

Then, fiber tractography was performed using MRtrix3 to explore white matter connectivity between the basal ganglia circuit and the rest of the brain. Whole-brain tractography was first conducted using the iFOD2 algorithm, which integrates fiber orientation distributions through a second-order probabilistic approach. A total of ten million streamlines were seeded randomly across the entire brain to serve as potential pathways for subsequent analysis.

Regions of interest (ROIs) were then defined based on fractional anisotropy (FA) images, which included 12 key regions of the basal ganglia circuit and brain regions showing significant inter-group differences determined by the FC one-way analysis of covariance (ANCOVA). These ROIs were carefully registered to each subject's T1 space using antsRegistration from ANTs, ensuring accurate anatomical alignment. Once registered, these ROIs were employed as inclusion regions for separate tractography runs, providing a targeted examination of the basal ganglia circuit connections. The tool *tckedit* was employed in this analysis. This allowed for the extraction of specific fiber bundles by specifying the start (seed) and end (target) points for each bundle, thereby isolating the tracts connecting defined regions of interest. The process used the default settings of *tckedit*, ensuring that the extracted streamlines accurately represented the direct structural connectivity between the targeted regions.

The final tract density maps were generated in native structural brain space using MRtrix3, with a threshold of 10 streamlines per voxel applied to exclude potentially spurious connections. After generating the tract masks, FSL was used to extract the mean FA value for each tract. The FA values were calculated from the preprocessed DTI data, which is a scalar value derived from DTI that quantifies the degree of directional dependence of water diffusion within tissues. Higher FA values indicate more anisotropic (directionally constrained) diffusion, which is typically seen in well-organized, dense fiber tracts, such as those found in white matter. In contrast, lower FA values suggest more isotropic (unrestricted) diffusion, often indicating areas with less structural integrity or regions where fibers cross or diverge. FA is particularly important in the study of white matter because it serves as a proxy for assessing microstructural integrity. Variations in FA can reveal subtle differences in the organization, density, and coherence of white matter tracts, which are critical for understanding brain connectivity.

### Statistical analyses

Statistics of demographic and clinical variables, GMV, fALFF, and FA values were performed using SPSS version 24.0. Initially, the Kolmogorov-Smirnov test was applied to determine the normality of the continuous variables. The demographic and clinical data conforming to normal distribution were analyzed using one-way ANOVA with post-hoc analysis and two-sample t-test. For the one-way ANOVA, the degrees of freedom were calculated as follows: between-group degrees of freedom (df₁) = *k* - 1, where k was the number of groups (*k* = 3 in this study); within-group degrees of freedom (df₂), which was equal to the degrees of freedom for post-hoc analysis, = N - k, where N was the total sample size. The two-sample t-test was used to compare the illness duration and inappropriate compensatory behavior frequency between the two patient groups, and its degree of freedom (df)= n_1_ + n_2_ – 2, where n_1_ and n_2_ were the sample sizes of the two patient groups, respectively. If GMV, fALFF, and FA values conformed to normal distribution, they were analyzed using one-way ANCOVA and post-hoc analysis with sex, age, education level, body mass index (BMI), and mean FD as covariates. For the one-way ANCOVA, the degrees of freedom were calculated as follows: between-group degrees of freedom (df₁) = *k* - 1, where k was the number of groups (*k* = 3 in this study); within-group degrees of freedom (df₂), which was also the degrees of freedom for post-hoc analysis, = N - k - p, where N was the total sample size and p was the number of covariates (*p* = 5 in this study). Non-normally distributed data were analyzed using the Kruskal-Wallis H test. In addition, the chi-square test was utilized to examine the sex variable. For the chi-square test, the degree of freedom was calculated as df = (number of rows - 1) × (number of columns - 1), and the degree of freedom of the chi-square test in this study is (3–1) × (2–1) = 2. Multiple comparison corrections for the group differences of GMV, fALFF, and FA values were performed using the Bonferroni correction, with statistical significance set at a corrected threshold of *p* < 0.05, and those results passing the correction were considered statistically significant. And for demographic and clinical data, statistical significance was considered at *p* < 0.05.

The statistical analyses of FC maps were performed using the general linear model in SPM12, with sex, age, education level, BMI, and mean FD as covariates. Firstly, the one-way ANCOVA (df₁ = 2, df₂ = N - 8) with sex, age, education level, BMI, and mean FD as covariates was applied to compare the differences in FC values among the three groups in the gray matter. Then the post-hoc analysis was performed to analyze the FC differences between each two groups using the areas with statistical differences identified by ANCOVA as mask. The post-hoc analysis was conducted using the two-sample t-test, with the degree of freedom (df) = n_1_ + n_2_ – 2, where n_1_ and n_2_ were the sample sizes of the two comparison groups, respectively. Then, the cluster-level FDR correction with a corrected threshold of *p* < 0.05 was employed for the multiple comparison corrections of results for ANCOVA and post-hoc analysis. And we utilized the DPABI software to extract FC values of regions with significant differences for subsequent analysis.

To explore the potential relationship between imaging features of the basal ganglia circuit and clinical characteristics, we performed the correlation analysis between the GMV/fALFF/FA/seed-based FC values in the regions with significant group differences and the clinical variables (disease duration, frequency of inappropriate compensatory behavior, DEBQ, EDI-BN, EAT-26, BDI, SAS scores) in patients with BN. The degrees of freedom for the correlation analysis were calculated as df = N - 2, where N was the sample size. Multiple comparison corrections for correlation analysis results were performed using the Bonferroni correction, with statistical significance set at a corrected threshold of *p* < 0.05.

In addition, to further assess the value of basal ganglia circuit imaging features as the risk factors for BN disease progression, we plotted the receiver operating characteristic (ROC) curves for the basal ganglia imaging features that showed significant differences between the two BN patient groups to evaluate their capacity of distinguishing mild BN patients from moderate-to-extreme BN patients. This step was performed in SPSS 24.0.

### Code availability

We have uploaded our codes of DTI data processing, white matter integrity property analysis and subsequent statistical analyses to GitHub for more transparent and reproducible science (https://github.com/guoweiwuorgin/the_basal_ganglia_circuit_underlie_the_severity_of_BN).

## Results

### Demographic and clinical characteristics

A total of 69 BN patients and 35 HCs were included in the study. Of these 69 patients, 34 were classified into the mild BN group and 35 were classified into the moderate-to-extreme BN group. Table 1 provides a comprehensive summary of demographic, clinical, and neuropsychological tests information of all participants. There were no statistically significant differences among the three subject groups in terms of age, sex, education, BMI, and mean FD (*p* > 0.05). The both BN patient groups exhibited the higher scores on the DEBQ (including restrained eating, emotional eating, and external eating), EDI-BN, EAT-26, BDI and SAS compared to the HC group (*p* < 0.05).

**Table 1:** Demographic and clinical characteristics of participants.

Characteristics	HCs (*n* = 35)	Mild BN (*n* = 34)	Moderate-to-extreme BN (*n* = 35)	*p* value
Age (year)	25.09 ± 2.44	24.38 ± 4.98	24.63 ± 5.93	0.82^a^
Sex (male/female)	2/33	3/31	2/33	0.84^b^
Education level (year)	17.20 ± 2.13	16.62 ± 2.15	16.40 ± 2.05	0.26^a^
BMI (kg/m^2^)	20.63 ± 1.83	21.20 ± 3.06	20.96 ± 4.01	0.74^a^
Mean FD	0.13 ± 0.05	0.12 ± 0.04	0.12 ± 0.06	0.61^a^
Illness duration (months)	NA	45.84 ± 41.25	56.94 ± 50.77	0.38^c^
Weekly inappropriate compensatory behaviors frequency (times/week)	NA	2.50 ± 0.75	9.37 ± 4.96	<0.001^c^^,^*
DEBQ-restrained eating (scores)	27.11 ± 7.72	41.12 ± 6.09	37.17 ± 6.18	<0.001^a^^,^*
DEBQ-emotional eating (scores)	27.17 ± 11.01	45.94 ± 11.24	49.03 ± 11.35	<0.001^a^^,^*
DEBQ-external eating (scores)	30.94 ± 5.05	35.09 ± 5.68	35.89 ± 6.26	0.001 a^,^*
EDI-BN (scores)	11.77 ± 4.24	30.82 ± 6.58	34.86 ± 5.30	<0.001^a^^,^*
EAT-26 (scores)	10.94 ± 8.43	43.82 ± 12.46	45.60 ± 10.26	<0.001^a^^,^*
BDI (scores)	4.25 ± 4.10	21.23 ± 8.29	25.17 ± 8.40	<0.001^a^^,^*
SAS (scores)	34.39 ± 9.29	52.50 ± 13.84	57.23 ± 11.56	<0.001^a^^,^*

a One-way ANOVA (df_1_ = 2 and df_2_ = 101).

b Chi-square test (df = 2).

c Two-sample *t*-test (df = 67).

⁎ The results were statistically significant (*p* < 0.05). Significant post-hoc analysis (*p* < 0.05): DEBQ-restrained eating (scores): HC < Mild BN, HC < Moderate-to-extreme BN, Moderate-to-extreme BN < Mild BN; DEBQ-emotional eating, DEBQ-external eating, EAT-26 and SAS (scores): HC < Mild BN, HC < Moderate-to-extreme BN; EDI-BN and BDI (scores): HC < Mild BN, HC < Moderate-to-extreme BN, Mild BN < Moderate-to-extreme BN. **Abbreviations:** HC, heathy control; BN, bulimia nervosa; BMI, body mass index; BDI, Beck Depression Inventory; BMI, body mass index; DEBQ, Dutch Eating Behavior Questionnaire; EAT, Eating Attitude Test; EDI-BN, bulimia subscale of the Eating Disorders Questionnaire; FD, frame displacement; NA, not applicable; SAS, Self-Assessment of Anxiety Scale.

There were no significant differences between the two groups of BN patients in terms of disease duration. The patients with moderate-to-extreme BN displayed a higher frequency of inappropriate compensatory behaviors compared to the patients with mild BN (*p* < 0.05). And the statistically significant differences were also detected between the two patient groups in DEBQ-restrained eating, EDI-BN, and BDI scores (*p* < 0.05).

### fALFF alterations in basal ganglia circuit among the three groups

**Fig. 2:**
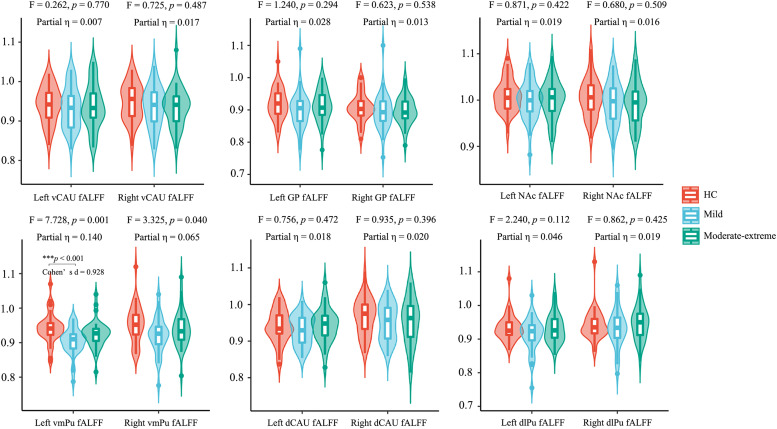
Differences in regional activity of the basal ganglia regions among the three groups. Significant group differences were found in the fALFF values of the left ventromedial putamen (*p* < 0.05, Bonferroni correction).

**Abbreviations:** fALFF, fractional amplitude of low-frequency fluctuation; vCAU, ventral caudate; GP, globus pallidus; NAc, nucleus accumbens; vmPu, ventromedial putamen; dCAU, dorsal caudate; dlPu, dorsolateral putamen.

Using the ANCOVA (df_1_ = 2, df_2_ = 96), we found significant group differences among the three groups in the fALFF values of the left vmPu (F [2, 96] = 7.728, *p* = 0.001, Partial η² = 0.140, Bonferroni corrected). Subsequent post-hoc analysis (df = 96) revealed that the mild BN group exhibited lower fALFF values of the left vmPu compared to the HC group (*p* < 0.001, Cohen's d = 0.936, Bonferroni correction). Conversely, no significant differences were observed between the moderate-to-extreme BN group and the HC group, nor between the two BN patient groups. No statistically significant group differences among the three groups were found for the fALFF values in other basal ganglia regions (Fig. 2).

### Seed-based FC alterations in basal ganglia circuit among the three groups

#### ANCOVA results

The ANCOVA analysis (df_1_ = 2, df_2_ = 96) revealed significant differences in seed-based FC among the three groups when utilizing the basal ganglia regions as seed points, which were detailed in Table 2 (cluster-level FDR correction with a corrected threshold of *p* < 0.05).

**Table 2:** Altered seed-based FC of basal ganglia circuit across three groups.

Seeds	Regions	Cluster size	F/t value	Peak MNI coordinates (mm)		
				x	y	*z*
*Three-group comparison*						
Left vCAU	Right ITG	141	12.55	57	−54	−15
	Left ITG	174	13.60	−48	−48	−21
	Left SMG	70	12.43	−60	−45	30
	Left PCUN	85	11.09	−3	−75	54
Left GP	Right SFGmed	74	13.15	9	42	36
Left NAc	Left OFC	156	22.84	−9	66	−9
Left vmPu	Right SFGmed	95	15.91	6	45	39
Left dCAU	Left SMG	91	12.04	−63	−39	39
Left dlPu	Left SFGmed	107	13.47	0	45	42
Right vCAU	Left ITG	94	11.53	−54	−36	−18
	Left SMG	70	12.68	−54	−42	24
Right NAc	Left OFC	140	18.12	−9	66	−9
Right vmPu	Right SFGmed	53	12.72	9	45	36
Right dCAU	Left SMG	134	15.91	−60	−42	36
Right dlPu	Left SFGmed	62	12.33	−3	42	42
*HC* vs*. mild*						
Left NAc	Left OFC	106	−5.61	−9	66	−9
Right NAc	Left OFC	122	−5.42	−3	60	−18
*HC* vs*. Moderate-to-extreme*						
Left vCAU	Right ITG	141	−5.55	57	−54	−15
	Left ITG	133	−4.77	−48	−66	−21
	Left SMG	57	−4.98	−60	−45	30
	Left PCUN	81	−5.01	−3	−75	54
Left GP	Right SFGmed	74	−5.41	9	45	36
Left NAc	Left OFC	145	−6.18	−9	66	−6
Left vmPu	Right SFGmed	95	−5.94	9	45	36
Left dCAU	Left SMG	89	−4.88	−57	−42	30
Left dlPu	Left SFGmed	107	−5.18	9	45	36
Right vCAU	Left SMG	37	−4.35	−57	−45	27
Right NAc	Left OFC	124	−5.19	−9	66	−9
Right vmPu	Right SFGmed	53	−5.13	9	45	36
Right dCAU	Left SMG	133	−5.95	−60	−42	36
Right dlPu	Left SFGmed	62	−4.89	−3	42	42
*Mild* vs*. Moderate-to-extreme*						
Left vCAU	Left ITG	142	−5.03	−54	−48	−15
	Left SMG	63	−4.50	−60	−33	36
Left GP	Right SFGmed	20	−4.01	9	39	33
Left vmPu	Right SFGmed	38	−4.13	3	45	39
Left dCAU	Left SMG	83	−4.32	−63	−36	36
Right vCAU	Left ITG	92	−5.10	−60	−48	−12
	Left SMG	69	−4.80	−54	−45	27
Right dCAU	Left SMG	43	−4.17	−60	−39	27

Three-group comparison was conducted using ANCOVA (df_1_= 2, df_2_ = 96).

Two-group comparisons (HC vs.Mild, HC vs. Moderate-to-extreme and Mild vs. Moderate-to-extreme) were performed using post-hoc analysis (df = 67, 68 and 67, respectively).

**Abbreviations:** FC, functional connectivity; vCAU, ventral caudate; ITG, inferior temporal gyurs; SMG, supramarginal gyrus; PCUN, precuneus; GP, globus pallidus; SFGmed, medial superior frontal gyrus; NAc, nucleus accumbens; OFC, orbitofrontal cortex; vmPu, ventromedial putamen; dCAU, dorsal caudate; dlPu, dorsolateral putamen.

#### Post-hoc analysis results of HCs vs. mild BN patients

**Fig. 3:**
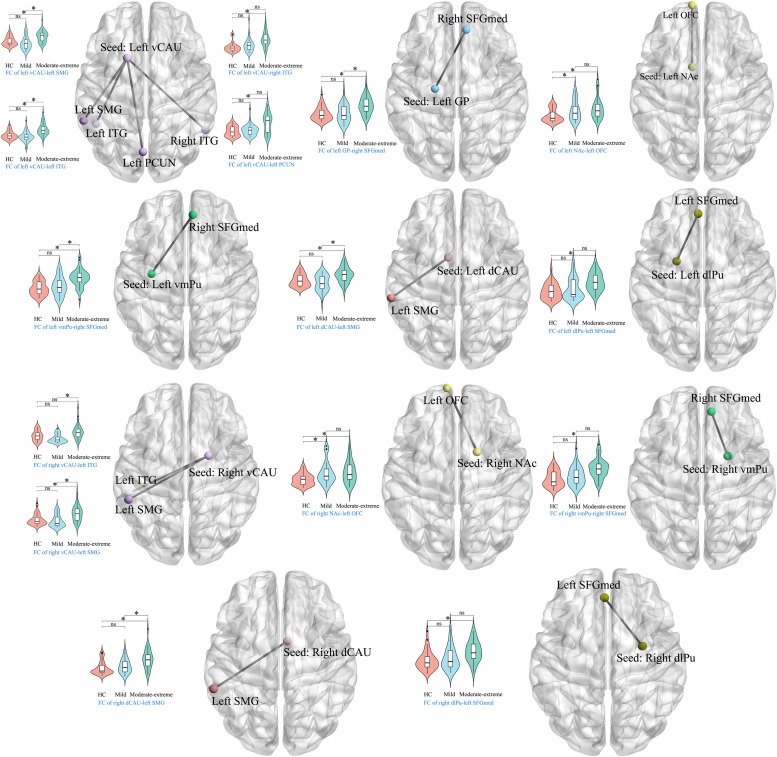
Seed-based FC post-hoc test results (cluster-level FDR correction with a corrected threshold of *p* < 0.05).

**Abbreviations:** FC, functional connectivity; vCAU, ventral caudate; ITG, inferior temporal gyurs; SMG, supramarginal gyrus; PCUN, precuneus; GP, globus pallidus; SFGmed, medial superior frontal gyrus; NAc, nucleus accumbens; OFC, orbitofrontal cortex; vmPu, ventromedial putamen; dCAU, dorsal caudate; dlPu, dorsolateral putamen

The post-hoc analysis (df = 67) between HCs and mild BN patients showed that compared to HCs, the mild BN patients showed increased FC between the bilateral NAc and the left orbitofrontal cortex (OFC) (cluster-level FDR correction with a corrected threshold of *p* < 0.05). We did not find significant group differences between mild BN patients and HCs in the seed-based FC of other basal ganglia regions (Table 2 and Fig. 3).

#### Post-hoc analysis results of HCs vs. moderate-to-extreme BN patients

The post-hoc analysis (df = 68) between HCs and moderate-to-extreme BN patients exhibited that compared to HCs, the moderate-to-extreme BN patients had higher FC between left vCAU and left inferior temporal gyrus (ITG)/right ITG/left supramarginal gyrus (SMG)/ left precuneus; between right vCAU and left SMG; between the left GP and the right medial superior frontal gyrus (SFGmed); between the bilateral NAc and the left OFC; between the bilateral vmPu and the right SFGmed; between the bilateral dCAU and the left SMG; and between the bilateral dlPu and the left SFGmed (cluster-level FDR correction with a corrected threshold of *p* < 0.05) (Table 2 and Fig. 3).

#### Post-hoc analysis result of mild BN patients vs. moderate-to-extreme BN patients

The post-hoc analysis (df = 67) between mild BN patients and moderate-to-extreme BN patients displayed that compared with the mild BN patients, the moderate-to-extreme BN patients exhibited increased FC between the bilateral vCAU and left ITG/left SMG; between the left GP and the right SFGmed; between the left vmPu and the right SFGmed; and between the bilateral dCAU and the left SMG (cluster-level FDR correction with a corrected threshold of *p* < 0.05) (Table 2 and Fig. 3).

### GMV alterations in basal ganglia circuit among the three groups

There were no significant differences in GMV in each basal ganglia region among the three groups, and detailed results are presented in Figure S1.

### White matter integrity alterations in basal ganglia circuit among the three groups

**Fig. 4:**
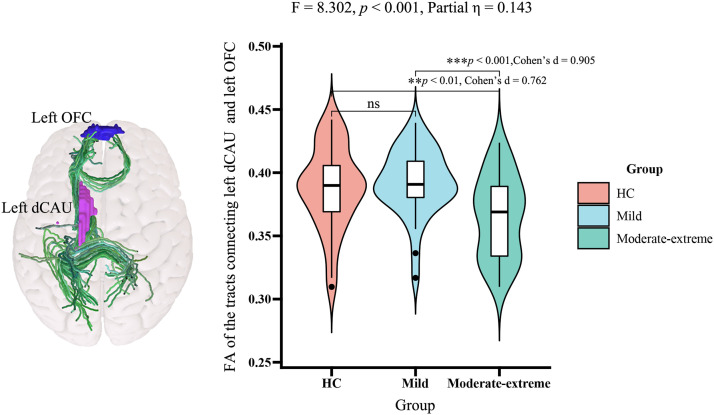
Anatomical diagram and FA values plots of the white matter tracts connecting the left dorsal caudate and left orbitofrontal cortex for the three groups (*p* < 0.05, Bonferroni correction).

**Abbreviations:** dCAU, dorsal caudate; OFC, orbitofrontal cortex; FA, fractional anisotropy.

In the ANCOVA (df_1_ = 2, df_2_ = 96), we identified significant group differences in the FA of the white matter tracts connecting the left dCAU and the left OFC among the three groups (F [2, 96] = 8.302, *p* < 0.001, Partial η² = 0.143, Bonferroni corrected). Subsequent post-hoc analyses (df = 96) showed that, compared to both HCs and mild BN patients, the moderate-to-extreme BN patients exhibited decreased FA in these white matter tracts (*p* < 0.05, Cohen's d = 0.762 and 0.905, Bonferroni correction). There were no significant group differences in FA of left dCAU-left OFC between HCs and mild BN patients. (Fig. 4). No other significant FA results were observed in our current study.

### Correlation between imaging features and clinical variables

**Fig. 5:**
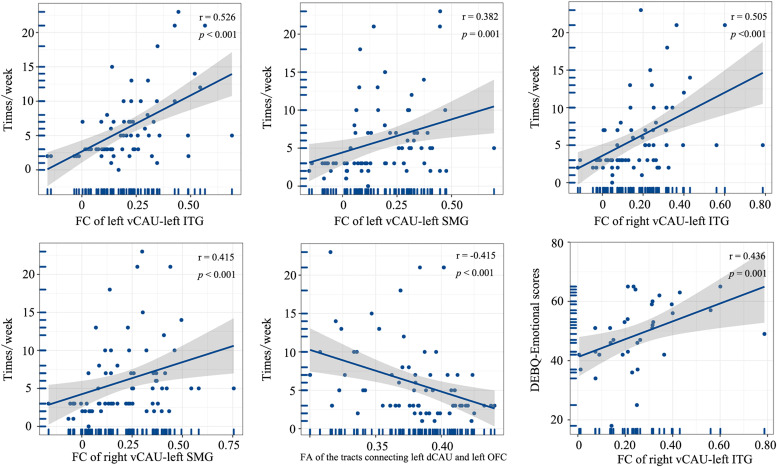
Correlations between brain imaging variables and clinical characteristics (*p* < 0.05, Bonferroni correction).

**Abbreviations:** times/week, inappropriate compensatory behavior episodes/week; vCAU, ventral caudate; ITG, inferior temporal gyurs; SMG, supramarginal gyrus; dCAU, dorsal caudate; OFC, orbitofrontal cortex; DEBQ, Dutch Eating Behavior Questionnaire.

In the entire patient group, the weekly frequency of inappropriate compensatory behaviors was positively correlated with the FC of left vCAU and left ITG, FC of left vCAU and left SMG, FC of right vCAU and left ITG, and FC of right vCAU and left SMG (r [67]= 0.526, *p* < 0.001; r [67]= 0.382, *p* = 0.001; r [67]= 0.505, *p* < 0.001; r [67]= 0.415, *p* < 0.001, Bonferroni corrected). And the FA of the white matter tracts connecting the left dCAU and left OFC exhibited a negative correlation with the weekly frequency of inappropriate compensatory behaviors (r [67] = −0.415, *p* < 0.001, Bonferroni corrected). In the moderate-to-extreme patient group, we identified a positive correlation between the DEBQ-emotional scores and FC of right vCAU and left ITG (r [33]= 0.436, *p* = 0.001, Bonferroni corrected) (Fig. 5), while such correlation was not found in the mild patient group.

### Classification of BN patients with different severity using basal ganglia circuit imaging features

**Fig. 6:**
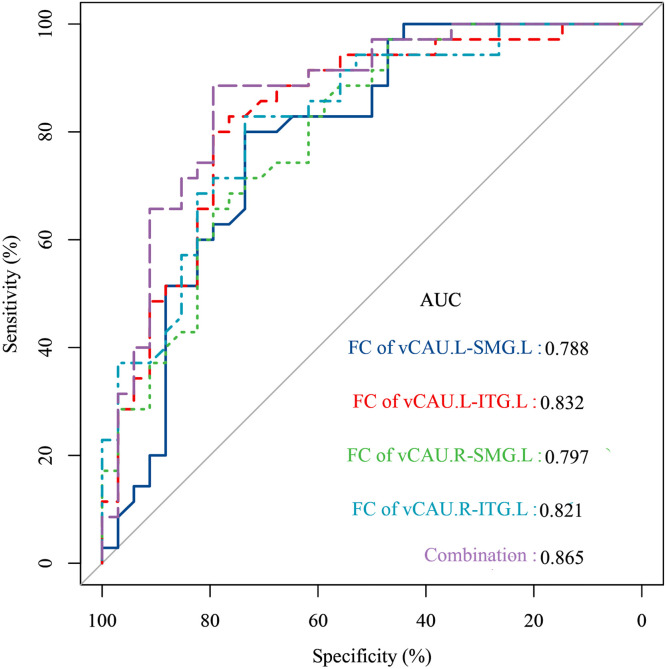
ROC curves of basal ganglia imaging properties as indicators of BN severity subtype differentiation.

**Abbreviations:** ROC, receiver operator characteristic; AUC, area under curve; vCAU, ventral caudate; SMG, supramarginal gyrus; ITG, inferior temporal gyurs; L, left; R, right.

Table 3, Fig. 6 and Figure S2 illustrate the efficacy of each basal ganglia circuit imaging feature and their combination in classifying mild and moderate-to-extreme patients. These measures include the areas under the curve (AUC) of the ROC curve, sensitivity, and specificity. Among these imaging features, seed-based FC of the basal ganglia regions demonstrated superior classification efficacy, as shown in Table 3 and Fig. 6. Specifically, the AUCs of the ROC were 0.778 for FC of left vCAU and left SMG, 0.832 for FC of left vCAU left ITG, 0.797 for FC of right vCAU and left SMG, and 0.821 for FC of right vCAU left ITG. The sensitivities of these FC were 0.880, 0.880, 0.657 and 0.829, and their specificities were 0.735, 0.794, 0.735 and 0.794, respectively. The AUC, sensitivity, and specificity for the combination of basal ganglia circuit imaging features were 0.865, 0.886 and 0.794 respectively. The ROC curves for the remaining image features and their efficiencies were detailed in Table 3 and Figure S2.

**Table 3:** Results of ROC curve analysis of basal ganglia imaging properties as indicators of BN severity subtype differentiation.

Basal ganglia regions’ imaging properties	AUC	Sensitivity	Specificity	*p*-Value	Cutoff
FC of vCAU.L-SMG.L	0.788	0.800	0.735	0.000*	0.130
FC of vCAU.L-ITG.L	0.832	0.800	0.794	0.000*	0.188
FC of vCAU.R-SMG.L	0.797	0.657	0.794	0.000*	0.242
FC of vCAU.R-ITG.L	0.821	0.829	0.735	0.000*	0.132
FC of GP.L-SFGmed.R	0.743	0.829	0.588	0.001*	0.183
FC of vmPu.L-SFGmed.R	0.741	0.800	0.706	0.001*	0.298
FC of dCAU.L-SMG.L	0.772	0.686	0.735	0.000*	0.214
FC of vCAU.R-SMG.L	0.757	0.600	0.824	0.000*	0.198
FA values of the white matter tracts connecting the left dCAU and left OFC	0.598	0.800	0.441	0.185	0.395
fALFF values of left vmPu	0.659	0.600	0.735	0.023*	0.917
Combination	0.865	0.886	0.794	0.000*	0.371

**Abbreviations:** ROC, receiver operator characteristic; AUC, area under curve; vCAU, ventral caudate; SMG, supramarginal gyrus; ITG, inferior temporal gyurs; GP, globus pallidus; SFGmed, medial superior frontal gyrus; vmPu, ventromedial putamen; vCAU, ventral caudate; dCAU, dorsal caudate; SMG, supramarginal gyrus; FA, fractional anisotropy; fALFF, fractional amplitude of low-frequency fluctuation; L, left; R, right.

## Discussion

Previous studies have shown that certain imaging features of the basal ganglia in patients with BN are associated with impulsivity, bulimic behavior, and inappropriate weight compensatory behaviors (Berner et al., 2019; Coutinho et al., 2015). However, it is still largely unknown whether and how the basal ganglia imaging features vary in BN patients with different severities. Notably, the severity of BN is closely tied to its treatment outcome, with mild patients reporting fewer eating disorder pathology symptoms and a greater likelihood of abstaining from maladaptive behaviors after receiving structured treatment compared to the moderate-to-severe patents (Gorrell et al., 2019; Smith et al., 2017). Comparing the basal ganglia imaging characteristics of mild BN patients with those of moderate-to-severe BN patients not only aids in elucidating basal ganglia imaging changes associated with BN disease progression but also contributes to the identification of imaging markers for BN disease progression, thus facilitating disease monitoring and treatment improvement. In short, our findings suggested that compared with HCs, basal ganglia circuit imaging alterations were subtle in patients with mild BN but were pronounced in patients with moderate-to-extreme BN, indicating that the basal ganglia circuit imaging alterations become more pronounced with increasing disease severity. The imaging alterations of basal ganglia circuit in moderate-to-extreme BN patients were mainly characterized by extensive functional network reorganization between the basal ganglia regions and the other brain regions, as well as specific impaired white matter integrity. Furthermore, our ROC curve analysis demonstrated that the functional network reorganization of the basal ganglia circuit is a valuable imaging feature for differentiating between mild and moderate-to-extreme BN patients.

### Common FC alterations in mild and moderate-to-extreme BN patient groups

The mild patients showed subtly abnormal seed-based FC, i.e., increased FC between the bilateral NAc and left OFC, which was also observed in moderate-to-extreme patients. NAc stands as a hub within the reward system, governing hedonic eating and modulating impulsive behaviors (Schuller & Koch, 2022). OFC plays a role in food intake control and satiety, and it may lead to disordered eating behavior through satiety and/or disturbances in the reward assessment for food stimuli (Monteleone et al., 2018; Plassmann, O'Doherty & Rangel, 2010; Rolls, 2008). Both regions are widely recognized for their key roles in the neuropathophysiology of eating disorders (Li et al., 2023; Walle et al., 2024; Wang et al., 2019). Several task-state functional MRI studies have demonstrated heightened OFC and NAc activity during food cue presentation (J. E. Lee, Namkoong & Jung, 2017; Weygandt, Schaefer, Schienle & Haynes, 2012), which could predict an individual's binge-eating symptom severity (G. J. Wang et al., 2011). The attenuated OFC activity in BN patients during food viewing and tasting has been reported in previous research, and this hypoactivity was associated with patients’ binge eating/purging episodes frequency (Frank, Reynolds, Shott & O'Reilly, 2011; Uher et al., 2004). Furthermore, scholars have previously discovered that an increased GMV in the NAc is associated with more frequent vomiting in BN patients (Schafer, Vaitl & Schienle, 2010). Our findings indicate that hyperconnectivity between two regions associated with the control of eating behavior may play a key role in the origin and persistence of this disease, as the increased FC between these two above-mentioned regions is present both in mild and moderate-to-extreme BN patients.

### FC alterations only in moderate-to-extreme patients

The primary alteration in the functional imaging of the basal ganglia circuit in moderate-to-extreme BN patients was its extensive functional network reorganization with other brain regions, whereas such alteration was very slight in the mild BN patients. In addition to the shared FC alterations in two patient groups, the moderate-to-extreme BN patients also showed enhanced FC between bilateral vCAU and left SMG/bilateral dCAU and left SMG/left GP and right SFGmed/bilateral vmPu and right SFGmed/bilateral dlPu with left SFGmed compared to HCs. In addition, the above FC and the FC between the bilateral vCAU and the left ITG of moderate-to-extreme patients compared to the mild were higher than those of mild BN patients. The CAU, GP (especially the ventral GP), Pu and SFGmed (part of the ventral medial prefrontal cortex [vmPFC]) are core regions involved in distinct temporal phases of rewarding processing as well as in the composition of the frontostriatal circuit (Haber & Knutson, 2010; Oldham et al., 2018; Silverman, Jedd & Luciana, 2015). Fischer et al. (2017) discovered that patients with BN exhibited hypoactivity in reward-related areas when receiving food cues, including areas such as the vmPFC and amygdala. The SMG, part of the inferior parietal lobule, has been proposed to be involved in negative self-perceptions in BN patients, and its activation was reduced when patients responded to words associated with negative emotions (Pringle, Ashworth, Harmer, Norbury & Cooper, 2011). The ITG is one of the key structures in the human brain for receiving and processing body image inputs (Bracci, Caramazza & Peelen, 2015; Mesulam, 1998). Impaired integrity of the white matter tracts connecting visually relevant areas of the temporal cortex was identified in patients with BN, and the severity of the impairment was positively correlated with the degree of concern about one's body shape (He, Stefan, Terranova, Steinglass & Marsh, 2016). As such, we conjectured that this abnormal hyperconnectivity, which is present only in moderate-to-extreme BN patients but absent in mild patients, may suggest that the abnormally reinforcing interactions within the reward processing regions and between the reward processing and negative self-perception areas/body perception regions may underlie the neural basis of disease progression. In other words, the progression of BN may have potential implications on the information exchange between reward-processing, self-perceptions, and vision-related regions. Furthermore, in the ROC curve analysis, we observed that the functional network reorganization of the basal ganglia circuit was a valuable imaging feature to differentiate between patients with mild and moderate-to-extreme BN, further suggesting that this imaging feature may be a marker of disease progression.

### Impaired white matter integrity only in moderate-to-extreme patients

Structural imaging variables differed less among the different severity patients compared to functional imaging variables. There were no GMV alterations in the entire BN patient group. Only moderate-to-extreme BN patients exhibited reduced FA in the white matter connecting the left dCAU to the left OFC, whereas mild patients had no significant white matter alterations. FA is used to quantitatively describe the degree of anisotropy in the diffusion of water molecules (He et al., 2016). The FA value is considered to represent the structural integrity of the white matter tracts, and the reduced FA suggests impaired white matter integrity (Chen et al., 2023). Frank et al. (Frank, Shott, Riederer & Pryor, 2016) have found that the structural connectivity of the white matter tract connecting the OFC and the striatum was increased in patients with BN and that this finding indicated a reduced integrity of this tract (Frank et al., 2016; Wang et al., 2015). Our findings suggest that the white matter integrity of the basal ganglia is preserved in patients with mild BN but may be partially compromised when the disease progresses.

Several potential limitations related to this study must be noted. First, though we established two groups of BN patients based on the purpose of our study, the sample size of our study is relatively small. Thus, future studies with larger samples are needed to verify the reproducibility of our findings. Second, the present study was a cross-sectional study with two patient subgroups from two samples, and although we controlled for some variables that may have had an impact on the results (e.g., disease duration), the results may still have been affected by other confounding factors, so future studies should track the longitudinal changes in basal ganglia circuit imaging features in BN patients from one sample over disease progression.

## Conclusion

The present study uncovers that the multimodal quantitative imaging alterations of the basal ganglia circuit are subtle in patients with mild BN and pronounced in patients with moderate-to-extreme BN, indicating a crucial role of basal ganglia circuit in the progression of BN. Among these multimodal imaging abnormalities, the extensive functional network reorganization between basal ganglia circuit and reward-processing, self-perceptions, and vision-related regions may be a potential imaging risk indicator for disease progression in patients with mild BN.

## Compliance with ethical standards

The research protocol was reviewed for compliance with the standards for the ethical treatment of human participants and approved by the Ethical Committee for Scientific Research at the hospital with which the authors are affiliated (Approval No 2021-P2–052–01).

## Research involving human participants

All ethical guidelines for human subjects’ research were followed.

## Informed consent

All participants provided written informed consents to participate in the study.

## Declaration of competing interest

The authors declare that they have no conflict of interest.

## Appendix Supplementary materials

Image, application 1
