# Supplementary material for: Multimodal quantitative magnetic resonance imaging alterations of the basal ganglia circuit underlie the severity of bulimia nervosa
Source: Int J Clin Health Psychol. 2025 Mar 13;25(1):100557. doi: 10.1016/j.ijchp.2025.100557 (PMC11953982; doi:10.1016/j.ijchp.2025.100557)
Supplement: Supplementary file 1 [file mmc1.docx]

**1. Supplementary methods and materials**

**1.1. Detailed parameters of different MRI sequences**

**1.1.1 High-resolution T1-weighted structural imaging**

The high-resolution T1-weighted structural images were collected in the sagittal position using a three-dimensional magnetization prepared rapid acquisition gradient-echo (MP-RAGE) sequence with the following parameters: repetition time (TR)/echo time (TE), 2530 ms/2.98 ms; inversion time (TI), 1100 ms; flip angle, 7°; data matrix, 256 × 256; voxel size, 1 × 1 × 1 mm^3^ ; field of view (FOV), 256 × 256 mm^2^ ; slice thickness/gap, 1.0 mm/1.0 mm; and number of slices, 192.

**1.1.2 Diffusion tensor imaging (DTI)**

DTI was acquired using a single-shot gradient-echo echo-planar imaging (EPI) sequence, and the detailed parameters were as follows: TR, 8500 ms; TE, 63 ms; data matrix, 128 × 128; voxel size 2 × 2 × 2 mm^3^ , FOV, 224 × 224 mm^2^ , nonzero b value, 1000 s/mm^2^ ; gradient directions, 64; slice thickness, 2 mm; and bandwidth, 2232 Hz/Px. We obtained 74 contiguous slices.

**1.1.3 resting-state functional MRI (**rs-fMRI**)**

The resting-state functional MRI images were acquired with the EPI sequence using the following parameters: TR/TE = 2,000 ms/30 ms; flip angle = 90°; matrix = 64×64; FOV = 224×224 mm^2^ ; slice thickness/gap = 3.5 mm/ 1 mm; slice number = 33; and time points = 240.

**1.2. Preprocessing of rs-fMRI data**

The main steps included the following: (1) slice timing was performed on the remaining 230 volumes after the first ten volumes were discarded (2) head movements were corrected for all subjects using a six-parameter rigid-body transformation, and subjects with head movements > 2.0 mm translation and 2.0° rotation in any direction were removed. We also calculated the mean frame displacement (FD) and used it as a covariate in the subsequent statistical analyses, as head movement would have potentially confounding effects on our results; (3) the T1-weighted images were then co-registered with the corresponding realigned functional images and segmented into: gray matter, white matter and cerebrospinal fluid (CSF); (4) we then normalized the generated rs-fMRI images to standard Montreal Neurological Institute (MNI) space using the same parameters as for structural image normalization and resampled them to a resolution of 3×3×3 mm^3^; (5) the converted images were then bandpass filtered in 0.01–0.08 Hz (this step was only applied to the data preprocessing of FC calculation, and was not applied to the data preprocessing of fALFF calculation); (6) regression of interference signals generated by Friston 24 head motion parameters, CSF signals, and WM signals were conducted; and (7) detrending and smoothing using a 6.0-mm FWHM Gaussian kernel were performed for each voxel.

**1.3. Preprocessing of T1-weighted structural data**

The main steps of this process were as follows: (1) the structural images were manually reoriented to place the anterior commissure at the origin and the anterior–posterior commissure in the horizontal plane if necessary; (2) a nonlinear deformation field was estimated to best cover the tissue probability map on the image of each individual subject; (3) then the generated images were segmented into gray matter, white matter and CSF areas using the unified standard segmentation option in SPM12; (4) after segmentation, the individual gray matter components were then normalized into standard MNI space using the Diffeomorphic Anatomical Registration through Exponentiated Lie Algebra (DARTEL) algorithm with a voxel size of 1.5 × 1.5 × 1.5 mm^3^; (5) The normalized gray matter components were modulated by a ‘non-linear only’ components derived from spatial normalization and were corrected with a bias-field correction to remove intensity non-uniformities; and (6) the modulated gray matter images were smoothed utilizing an 8-mm fullwidth half-maximum (FWHM) Gaussian kernel.

**2. Supplementary Figures**

**
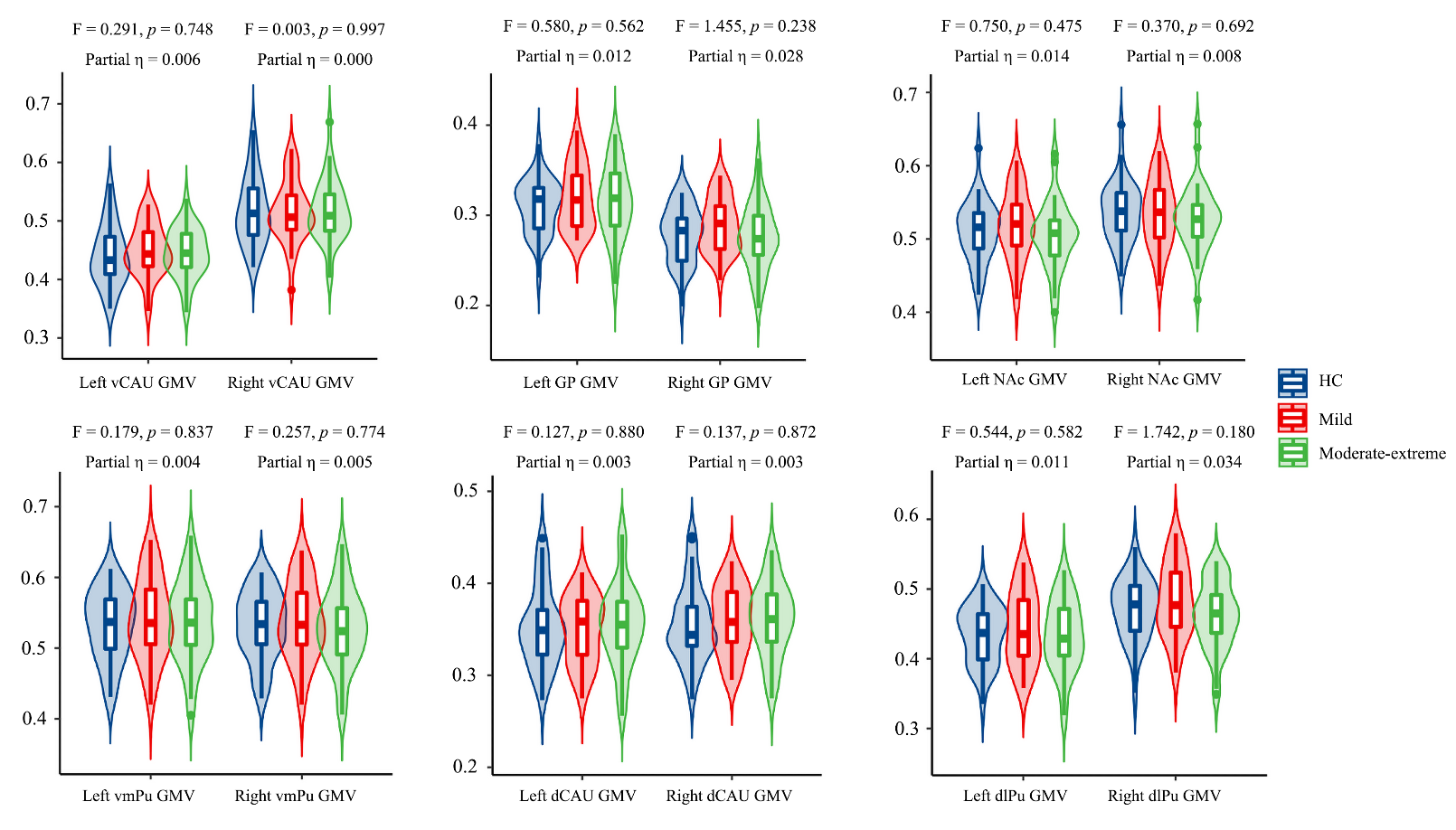
****Figure S1.** Plots of GMV values in the regions of basal ganglia circuit for the three participant groups. **Abbreviations:** GMV, gray matter volume; vCAU, ventral caudate; GP, globus pallidus; NAc, nucleus accumbens; vmPu, ventromedial putamen; dCAU, dorsal caudate; dlPu, dorsolateral putamen.


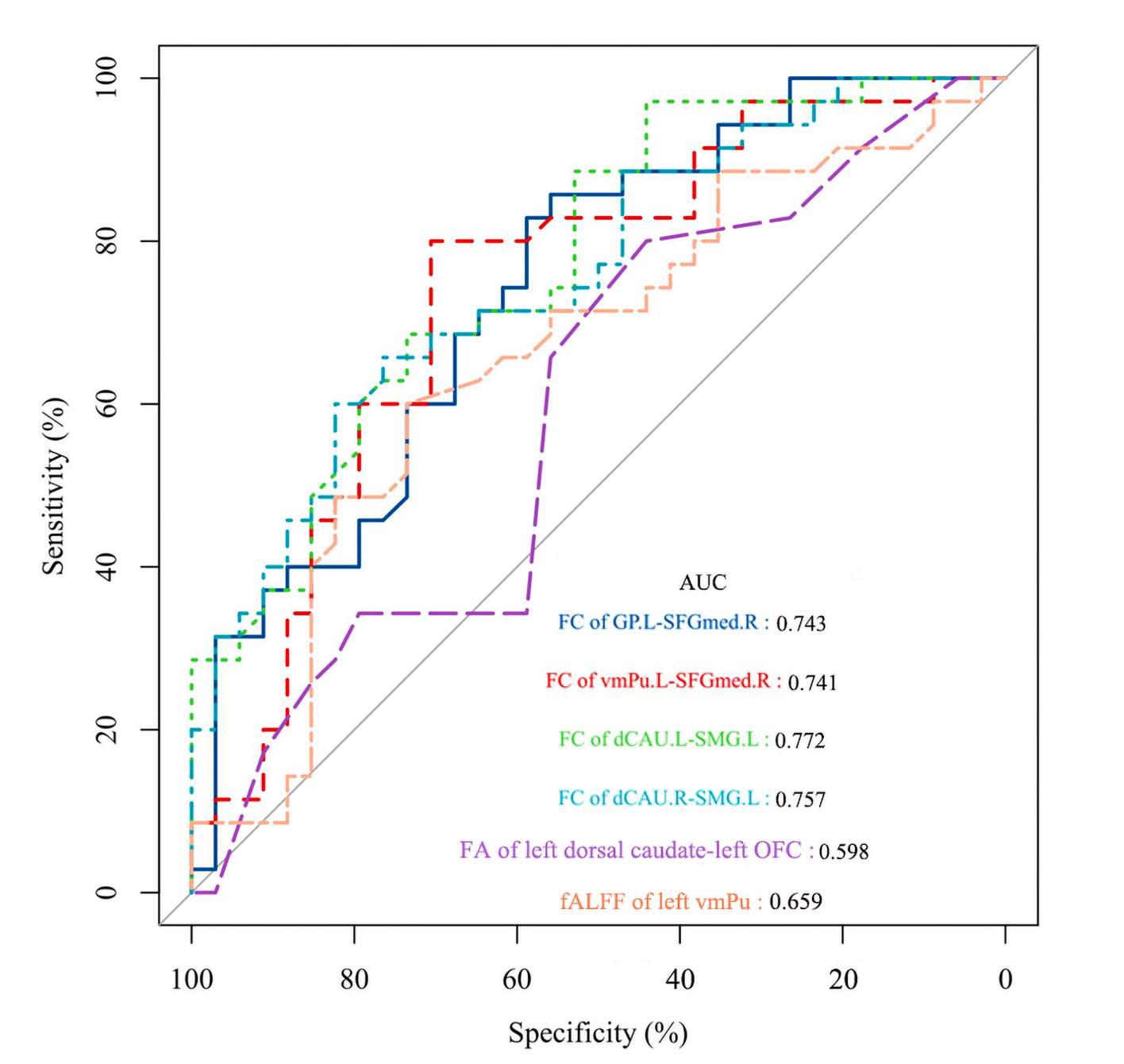


**Figure S2.** Additional ROC curves of basal ganglia imaging properties as indicators of BN severity subtype differentiation.

**Abbreviations:** ROC, receiver operator characteristic; BN, bulimia nervosa; AUC, area under curve; GP, globus pallidus; SFGmed, medial superior frontal gyrus; vmPu, ventromedial putamen; vCAU, ventral caudate; dCAU, dorsal caudate; SMG, supramarginal gyrus; FA, fractional anisotropy; fALFF, fractional amplitude of low-frequency fluctuation; L, left; R, right.
